# Characterizing and Implementing the Hamamatsu C12880MA Mini-Spectrometer for Near-Surface Reflectance Measurements of Inland Waters

**DOI:** 10.3390/s24196445

**Published:** 2024-10-05

**Authors:** Andreas Jechow, Jan Bumberger, Bert Palm, Paul Remmler, Günter Schreck, Igor Ogashawara, Christine Kiel, Katrin Kohnert, Hans-Peter Grossart, Gabriel A. Singer, Jens C. Nejstgaard, Sabine Wollrab, Stella A. Berger, Franz Hölker

**Affiliations:** 1 Department of Engineering, Brandenburg University of Applied Sciences, 14770 Brandenburg an der Havel, Germany; 2 Plankton and Microbial Ecology, Leibniz Institute of Freshwater Ecology and Inland Fisheries, 16775 Stechlin, Germany; igor.ogashawara@igb-berlin.de (I.O.); christine.kiel@igb-berlin.de (C.K.); katrin.kohnert@igb-berlin.de (K.K.); hanspeter.grossart@igb-berlin.de (H.-P.G.); jens.nejstgaard@igb-berlin.de (J.C.N.); sabine.wollrab@igb-berlin.de (S.W.); stella.berger@igb-berlin.de (S.A.B.); 3 Community and Ecosystem Ecology, Leibniz Institute of Freshwater Ecology and Inland Fisheries, 12587 Berlin, Germany; gschreck@gmx.de (G.S.); franz.hoelker@igb-berlin.de (F.H.); 4 Department of Monitoring and Exploration Technologies, Helmholtz Centre for Environmental Research-UFZ, 04318 Leipzig, Germany; jan.bumberger@ufz.de (J.B.); bert.palm@ufz.de (B.P.); paul.remmler@ufz.de (P.R.); 5 Research Data Management—RDM, Helmholtz Centre for Environmental Research-UFZ, 04318 Leipzig, Germany; 6 German Centre for Integrative Biodiversity Research (iDiv) Halle-Jena-Leipzig, 04103 Leipzig, Germany; 7 Institute of Biochemistry and Biology, Potsdam University, 14469 Potsdam, Germany; 8 Berlin-Brandenburg Institute of Advanced Biodiversity Research (BBIB), 14195 Berlin, Germany; 9 Department of Ecology, University of Innsbruck, 6020 Innsbruck, Austria; gabriel.singer@uibk.ac.at; 10 Institute of Biology, Freie Universität Berlin, 14195 Berlin, Germany

**Keywords:** mini-spectrometers, proximity sensing, water-leaving reflectance, sensor networks, freshwater monitoring, environmental photonics, compact spectro-radiometers

## Abstract

In recent decades, inland water remote sensing has seen growing interest and very strong development. This includes improved spatial resolution, increased revisiting times, advanced multispectral sensors and recently even hyperspectral sensors. However, inland waters are more challenging than oceanic waters due to their higher complexity of optically active constituents and stronger adjacency effects due to their small size and nearby vegetation and built structures. Thus, bio-optical modeling of inland waters requires higher ground-truthing efforts. Large-scale ground-based sensor networks that are robust, self-sufficient, non-maintenance-intensive and low-cost could assist this otherwise labor-intensive task. Furthermore, most existing sensor systems are rather expensive, precluding their employability. Recently, low-cost mini-spectrometers have become widely available, which could potentially solve this issue. In this study, we analyze the characteristics of such a mini-spectrometer, the Hamamatsu C12880MA, and test it regarding its application in measuring water-leaving radiance near the surface. Overall, the measurements performed in the laboratory and in the field show that the system is very suitable for the targeted application.

## 1. Introduction

Inland waters are hotspots of biodiversity and of great importance for humans as sources of food and water as well as for leisure activities [1]. The anthropogenic pressure on freshwater ecosystems is high because humans live in close proximity to freshwater bodies [2]. Climate warming and more extreme weather events put further strains on these important ecosystems. Eutrophication and harmful algal blooms [3,4] are issues that can potentially be monitored at a global scale by remote sensing [5,6,7] with multispectral [8,9] and hyperspectral imaging [10] systems, with the latter still being limited in coverage but more and more sensors becoming available [11,12]. One issue hampering the global application of inland water remote sensing is cloud coverage [13] and its low temporal resolution compared to high-frequency “in situ” monitoring concepts [14] using sub-surface sensors. Another challenging aspect of inland water remote sensing is atmospheric correction, which often requires additional ground-based reflectance measurements with ground-based (mostly handheld) spectro-radiometers [8,9,15]. This manual ground-truthing is labor- and cost-intensive and cannot be implemented on a regular basis [9,13,16]. Furthermore, inland waters have a higher diversity in terms of optical parameters, even at small spatial scales [9]. Therefore, there is an increased demand for large-scale ground-based multi- or hyperspectral sensor networks to support inland water remote sensing. Ideally, these should be self-sufficient as well as low-cost but also robust and not maintenance-intensive. Low-cost mini-spectrometers enable affordable and rigid spectro-radiometer systems [17]. Such ground-based near-surface hyperspectral sensors also have other applications in environmental sciences, for example, determining soil moisture [18], vegetation monitoring [19] and gas detection [20]. While for most of the aforementioned applications, relatively expensive hardware is used [9,19], recent advances in sensor technology have enabled low-cost sensors that are promising for realizing large sensor networks [17], as demonstrated for air pollution monitoring [21,22].

In this work, we test a low-cost hyperspectral sensor, the Hamamatsu C12880MA mini-spectrometer, and assess its applicability for reflectance measurements for inland waters. We describe the hardware and software implementation of a prototype for radiance measurements (total costs < USD 200). Furthermore, the idea of the ground-based sensor network is outlined.

## 2. Ground-Based Sensor Network for Near-Surface Sensing of Inland Waters

**Figure 1:**
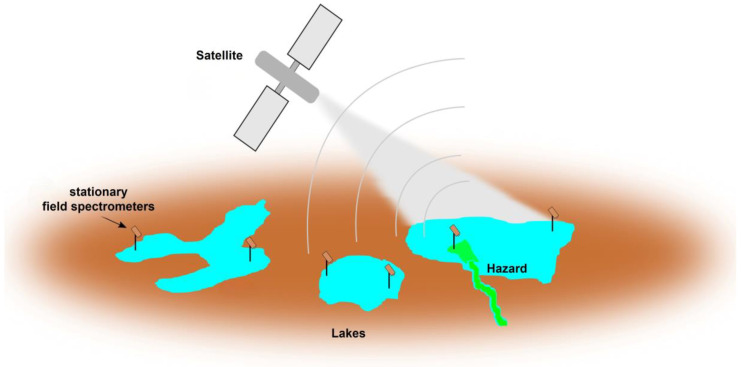
Schematic drawing of a network of near-surface optical sensors positioned on inland waters (for example, lakes shown in blue with a potential pollution hazard shown in green). The sensor network complements remote sensing techniques. Here, the sensors deliver essential ground-truth data to improve the performance of space-born sensors. On top of that, the ground-based network can fill gaps between revisiting times, with longer periods of cloud cover. While typically having lower data quality than underwater sensors, the optical sensors require less maintenance and, when produced at low costs, can in principle be operated in higher quantities, i.e., on larger scales.

Figure 1 shows a sketch of a ground-based sensor network of hyperspectral near-surface sensors installed at several lakes. Without cloud coverage, the satellite will cover the entire area. For example, at the moment, the Sentinel-2 tandem provides the best compromise between spatial resolution and spectral bands that are useful for inland water remote sensing. Its revisiting time is 5 days near the equator and can be less than that at higher latitudes when different viewing angles are also considered [9]. In the proposed network, the data from the ground-based sensors can be utilized for advanced atmospheric correction tailored to individual sites, which might be necessary to acquire better accuracy in bio-optical modeling [9]. Furthermore, the ground-based near-surface sensors will also acquire valuable, but spatially limited, data in between the satellite overpasses and also under cloud cover [13]. On the other hand, it is clear that not every water body can be monitored with in situ sensors and that there are spatial heterogeneities within lakes that can only be detected with remote sensing techniques [9].

Often, underwater multi-parameter sondes are used to obtain such high-frequency data for inland water systems, and, particularly in profiling underwater sensors, they provide additional depth-resolved information [23,24]. However, these systems are very costly and usually require a high level of maintenance, particularly in productive inland waters when, e.g., the overgrowth of algae becomes a problem.

Here, we point out that simple and miniaturized optical sensors do not require sophisticated infrastructure and have dramatically reduced maintenance requirements compared to underwater sensors [23,24]. Thus, more sensors can be distributed and more water bodies can be potentially monitored with these sensors compared to costly maintenance-intensive equipment.

Recently, even the power of a small sensor network was demonstrated with three optical sensors distributed on just three lakes over large distances where heat wave impacts could be observed [25]. In that study, the authors used the WISP-3 portable water spectrometer (Water Insight, Ede, The Netherlands), which is based on several commercial spectrometers [26,27].

## 3. Materials and Methods

### 3.1. The Optical Sensor

**Figure 2:**
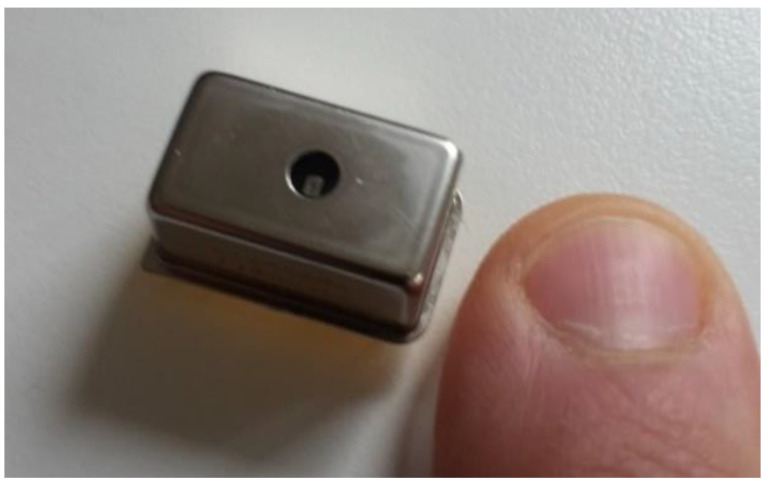
The optical sensor, Hamamatsu C12880MA (image, A. Jechow).

The employed optical sensor is a Hamamatsu C12880MA (Hamamatsu Photonics K.K., Hamamatsu, Japan) mini-spectrometer (Figure 2). This sensor was chosen due to its compact size, low cost, rigid housing and optical parameters. This device features a high sensitivity and a nominal spectral band ranging from 340 to 850 nm with a wavelength resolution of 15 nm. The CMOS (Complementary Metal–Oxide–Semiconductor) sensor in combination with a miniaturized MEMS (Micro-Electrical Mechanical System) concave grating and an entrance slit allows for a compact size (20.1 mm × 12.5 mm × 10.1 mm) with an air- and watertight housing that offers high stability against moisture and dust.

The sensor requires a 5 V voltage source and a clock (CLK) between 200 kHz and 5 MHz, which is output by the sensor as a trigger (TRG) signal with a time shift. A pulse at the start (ST) pin starts a measurement, and, at the same time, the duration of the pulse determines the integration time. With the falling edge of the start pulse, the internal shift register starts to work, and the measured values are transmitted synchronously with the rising edges of the TRG signal. The measured values are output as analog voltage levels between 0 and 5 volts. When all values have been transmitted, the sensor sets the EOS to the high level for two clock pulses, which ends the measurement. For more details on the clock implementation, see the Supplementary Materials. See also Table 1.

### 3.2. Hardware and Software Implementation

**Figure 3:**
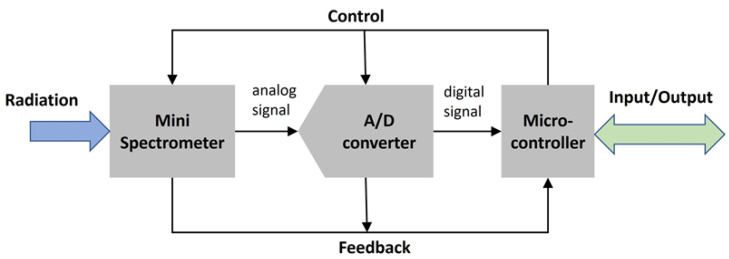
Sketch of the sensor system principle.

Figure 3 shows the principal sketch of the sensor system, where the analog signal of the sensor is converted using an analog-to-digital converter (ADC) and a micro-controller for the control and readout of the sensor platform. As the system should perform self-sufficiently on a lake, power consumption has to be taken into account when selecting the ADC and the microcontroller, in addition to the resolution and sampling rate.

The resolution is given by a readout noise of 1.3 mV (manufacturer), which results in an absolute minimum of 12 bit, but a reasonable lower limit of 16bit should be considered. There is also a trade-off as higher resolutions are only possible at the expense of lower sampling rates. A high sampling rate also requires higher processing speeds and thus promotes increased energy consumption. As a compromise, and considering the requirement of economic efficiency, the sampling rate is set to 1 MSPS. Consequently, the maximum possible sensor clock frequency is limited to 1 MHz.

**Figure 4:**
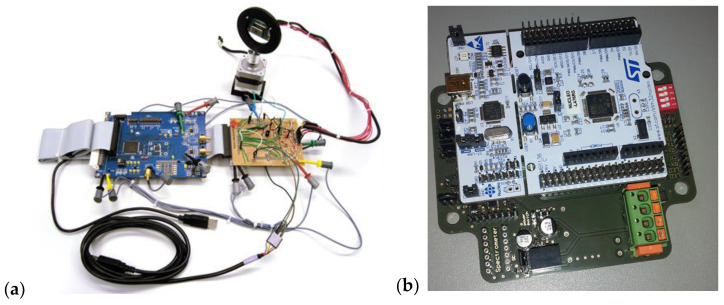
(**a**) Prototype electronics with laboratory setup and (**b**) microcontroller on PCB board (images: B. Palm, A. Jechow).

For initial fast prototyping, development boards were used for the ADC and the micro-controller (Figure 4a). The ADC development board (EVAL-AD7671CBZ, Analog Devices Inc., Norwood, MA, USA) integrates a 16-bit, 1 MSPS, successively approximating ADC chip and provides a 16-bit-wide parallel interface for reading the digital data. After configuring the board, analog input signal levels between 0 and 5 V can be digitized. The levels of the logic signals are adapted to the GPIO level of the µC and correspond to 0 V (low) and 3.3 V (high). The micro-controller development board (NUCLEO-L476RG, STMicroelectronics N.V., Geneva, Switzerland) is based on an ultra-low-power ARM Cortex-M4 32-bit CPU with FPU and can be operated with a maximum clock frequency of 80 MHz. Furthermore, numerous periphery functions, such as various interfaces, FLASH and SRAM memory and timers, are available (see the Supplementary Materials for details).

The firmware was written in the C programming language and was developed with the integrated development environment AC6 (System Workbench for STM32, version 1.15.0). Furthermore, the generator for initialization code CubeMX (STM32CubeMX, Version 4.22.0, STMicroelectronics N.V.) was used, as well as a hardware abstraction layer (HAL) and libraries provided by the manufacturer. The program flow can be summarized as a recurring sequence of waiting to receive an external command, executing the associated action, and handling any errors. Commands are received by the Universal Asynchronous Receiver Transmitter (UART) module and can be delayed by changing the CPU to sleep mode. The actions can be divided into set parameters, send parameters, start measurement(s) and stop measurement(s). An extended program flow chart is shown in the Supplementary Materials, where the main program flow, the measurement routine, the communication with the system and the protocol are also explained. The firmware developed for the measurement system was published under the EUPL 1.2 license at https://github.com/Helmholtz-UFZ/MiniSpecFirmware (accessed on 29 September 2024) and can be reused accordingly.

After successful testing, PCB boards were manufactured and a housing constructed (Figure 4b). In order to operate the system self-sufficiently over longer periods of time, the energy consumption of the system should be as low as possible. For this purpose, one can switch off consumers that are not used or switch them to suitable low-power modes. This is feasible since the ADC can switch to a power-down mode and the level converters provide inputs. The micro-controller already uses sleep mode when waiting for commands, but it can be put into an even ‘deeper’ low-power mode, where its current consumption is 112 nA, as opposed to 29.6 mA in sleep mode. Wake-up times increase with deeper low-power modes but are insignificant as they are well below one millisecond for all modes, thus delaying an initiated measurement only imperceptibly. In sleep mode, and especially during execution, it is mainly the system clock that determines the energy consumption. The sensor was wavelength- and sensitivity-calibrated in the laboratory, including dark current correction. The circuit design of the PCB board was published under the EUPL 1.2 license at https://github.com/Helmholtz-UFZ/MiniSpecHardware (accessed on 29 September 2024) and can be reused accordingly.

### 3.3. Measurement Setup

Figure 4a shows the measurement setup for the angular characterization. The detailed measurement routine and setup are provided in the Supplementary Materials.

## 4. Results

### 4.1. Sensor Characterization in the Laboratory

**Figure 5:**
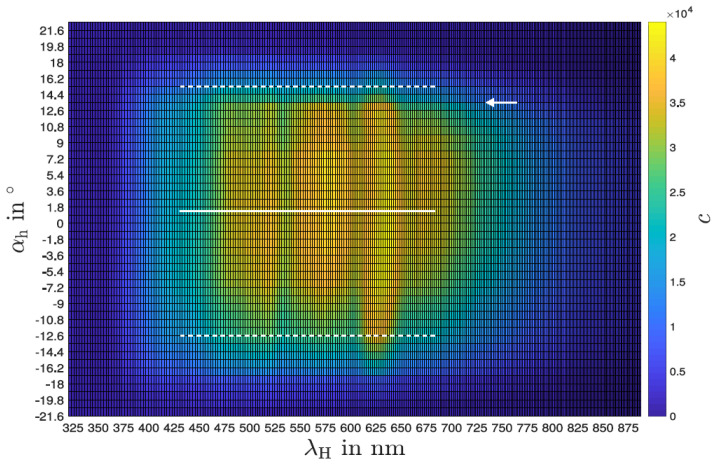
Sensitivity as a function of angle with the slit in the horizontal position for all wavelengths individually.

**Figure 6:**
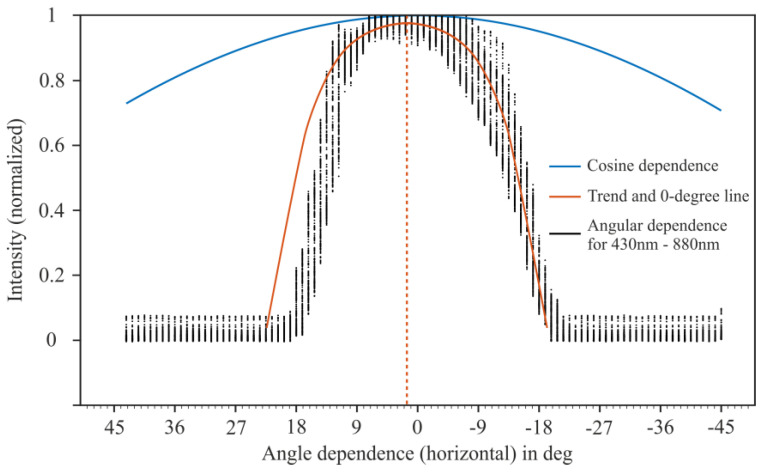
Sensitivity as a function of angle with the slit in the horizontal position for all wavelengths integrated (orange solid line, trend line).

Figure 5 shows the wavelength spectrum of the sensor for different angles with the slit in the horizontal position (“experiment E-2”; see the Supplementary Materials). A sharp drop in intensity over all wavelengths can be seen from +13°. Also, the whole dataset is not perfectly symmetrical around 0°, which could be a systematic error in our setup. Potentially, there could have been step losses in the stepper motor during the measurement. The small space in the darkening box and the hardly avoidable inertia and stiffness of the sensor lines could be responsible for this loss in steps. Despite that, the intensity drop with the increase in the angle, in the positive as well as negative direction, is clearly visible. Integrating the angle-specific intensity over all wavelengths to the maximum value (see Figure 6) confirms the small offset but also gives rise to a horizontal opening angle of less than 30°.

**Figure 7:**
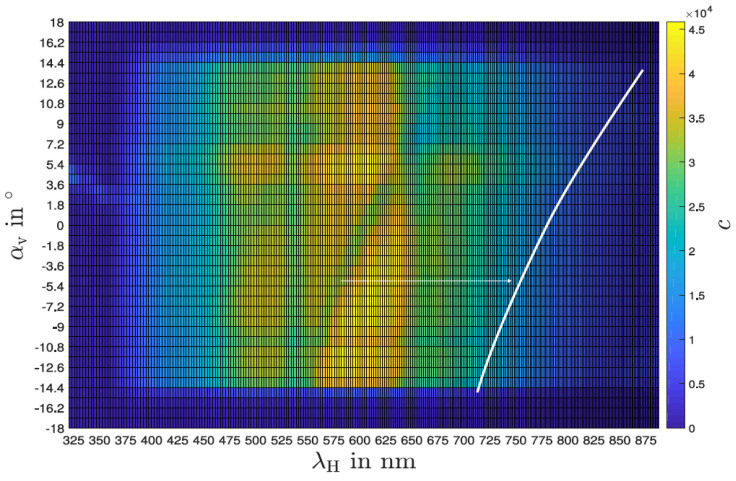
Sensitivity as a function of angle with the slit aligned vertically for all wavelengths individually in a density plot.

**Figure 8:**
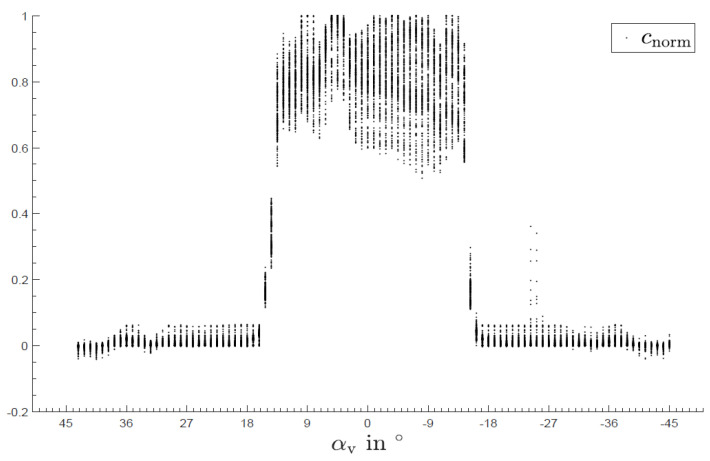
Sensitivity as a function of angle with the slit aligned vertically for all wavelengths integrated.

The wavelength-resolved angular behavior with the slit vertically aligned is shown in Figure 7. There, several artifacts can be seen, most notably an oblique line-like progression of an intensity attenuation through the points (−10°, 550 nm) and (12°, 700 nm). The reason for this artifact is difficult to identify; it could be extrinsic, underlying the examination setup, or intrinsic, i.e., sensor- or platform-related. A random deviation can be excluded in any case since a clear dependence between angle and wavelength can be seen. This dependence also appears to be almost linear, and furthermore independent of direction and time because the angles were measured from positive as well as from negative starting points. In practical use, there is generally no directional parallel radiation, but rather diffuse radiation, with different intensities being emitted from different directions. Therefore, it can be assumed that the observed effect is less pronounced during field use than under laboratory conditions, especially because the sensor only shows relative intensity differences. An integrated plot is shown in Figure 8 with the normalized intensity of all wavelengths over the angles. There, the vertical opening angle is between 26° and 30°.

**Figure 9:**
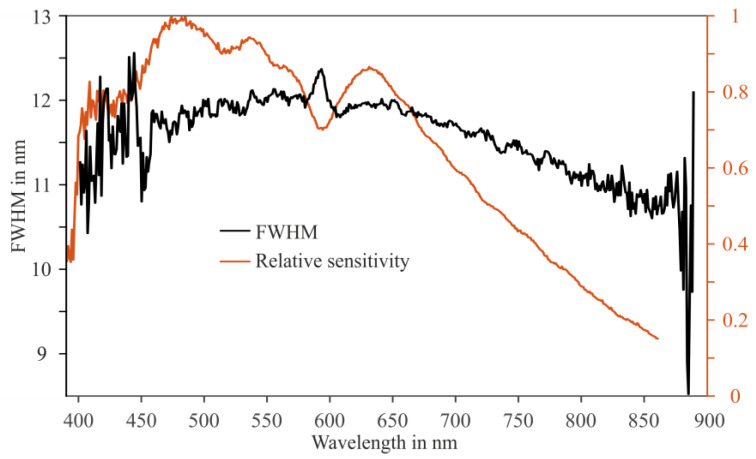
Relative responsivity normalized to the peak (solid orange line) and spectral resolution (solid black line) of the micro-spectrometer as a function of the wavelength. FWHM—full width at half maximum.

The wavelength-resolved normalized responsivity and the wavelength resolution (FWHM) are shown in Figure 9. The spectral resolution ranges between 11 nm and 12 nm, which is sufficient for application in inland water spectroscopy. The overall judgment of the sensor characterization in the laboratory is that it meets the required specifications.

### 4.2. Field Test

#### 4.2.1. Field Test at Lakes

**Figure 10:**
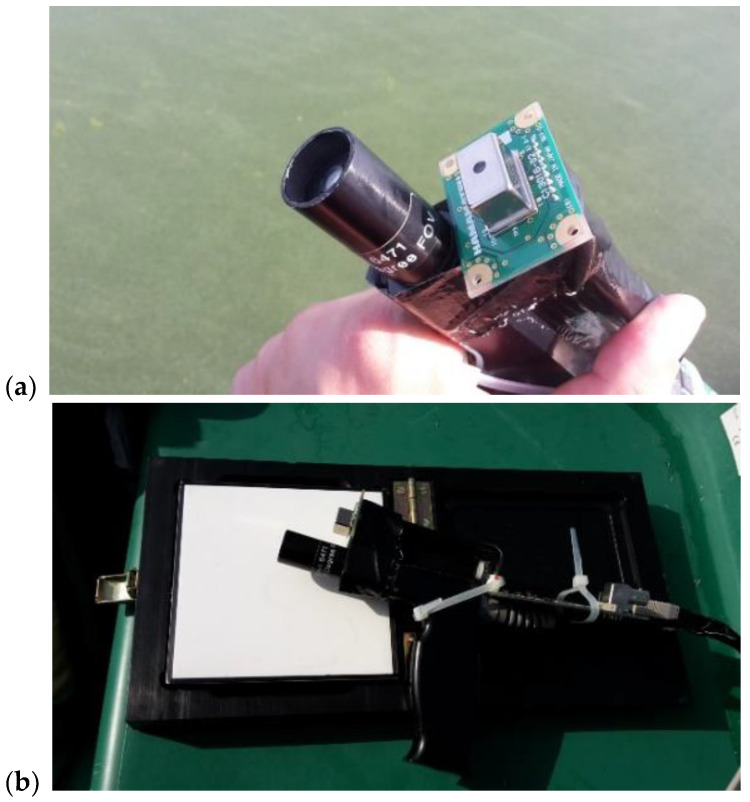
Mini-spectrometer tested in the field: (**a**) ASD field spec optics and Hamamatsu mounted next to each other; (**b**) ASD and Hamamatsu with reflectance plate (Spectralon, 99%, 10″) on the boat (Images, A. Jechow).

An early version of the low-cost spectrometer system prototype was tested on two different lakes during a remote sensing calibration and validation campaign. To avoid pointing errors due to boat movement or human interference, the sensor head was mounted directly in the vicinity of the field spectrometer, ASD field spec 4 (Analytik Ltd., Cambridge, UK), and measurements were obtained in parallel (Figure 10a). At both sites, the spectro-radiometer systems were used in the Mobley architecture to obtain remote sensing reflectance (R_RS_) [9,15,28,29]. A large-area high-reflectance standard (Spectralon, 10″, 99%, LABSPHERE, North Sutton, NH, USA) was used (Figure 10b). The properties of the two spectro-radiometer systems are listed in Table 2.

**Figure 11:**
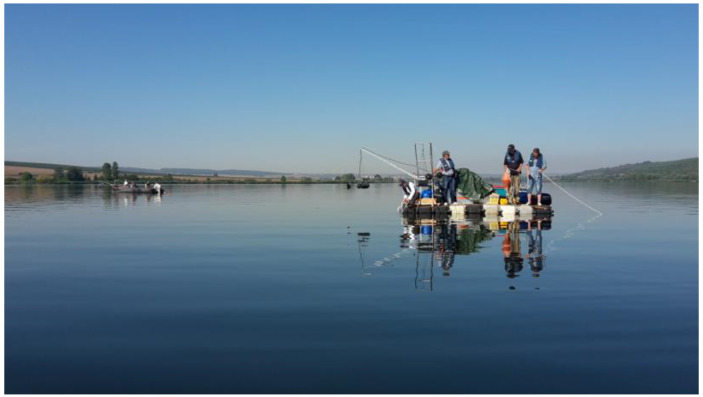
Measurements during the Remote Sensing Campaign at Lake Süßer See (Image, A. Jechow).

**Figure 12:**
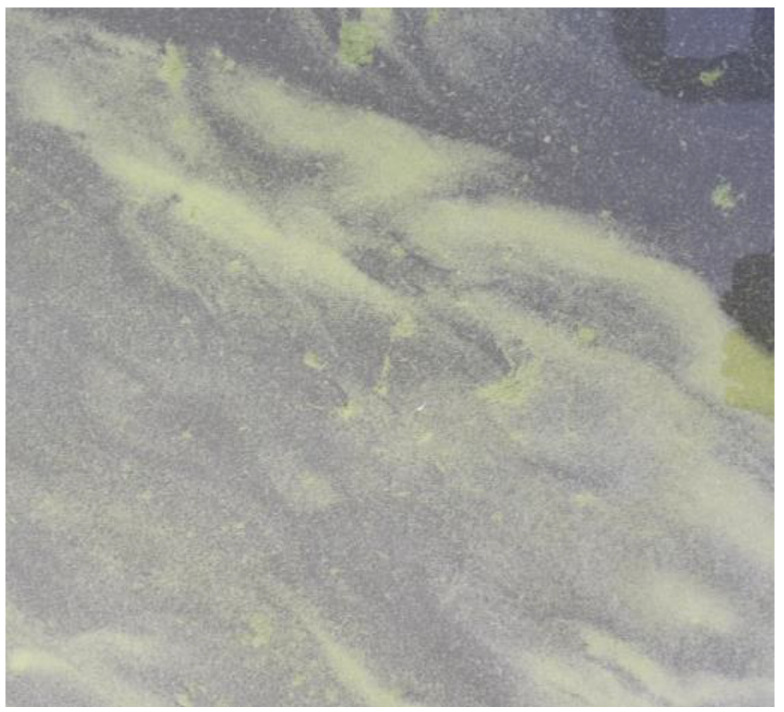
Image of the water surface during the measurements during the Remote Sensing Campaign at Kelbra dam (Image, A. Jechow).

Measurements were performed at two lakes within the Inland Water Remote Sensing Validation Campaign 2017 [30]. Lake Süßer See was sampled on 29 August 2017, within the central intercalibration campaign [31]. On this day, the sky was perfectly clear, and there was almost no wind. This resulted in a homogeneous water surface. Furthermore, there was no apparent algal bloom (Figure 11). Kelbra reservoir [32] was sampled with a smaller sub-team on 30 August 2017, with an apparent phytoplankton bloom with floating algae clusters near the surface (Figure 12). The weather was also clear sky and no wind, resulting in an even and flat water surface.

**Figure 13:**
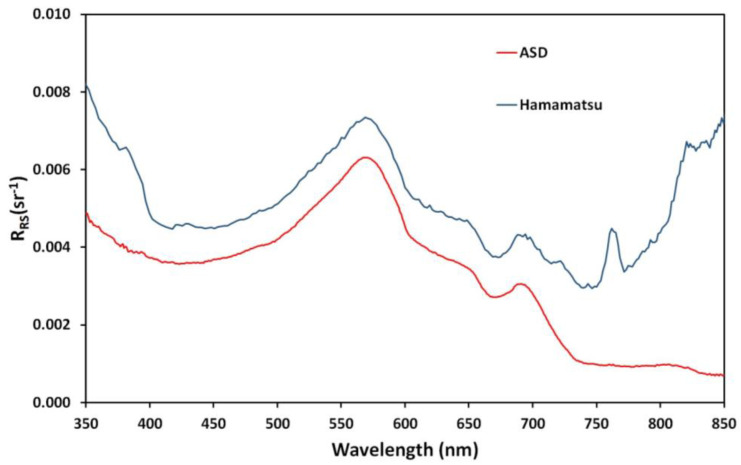
Remote sensing reflectance (R_RS_) obtained at Lake Süßer See using the two spectro-radiometer systems, with the red line representing ASD and the blue line the mini-spectrometer.

**Figure 14:**
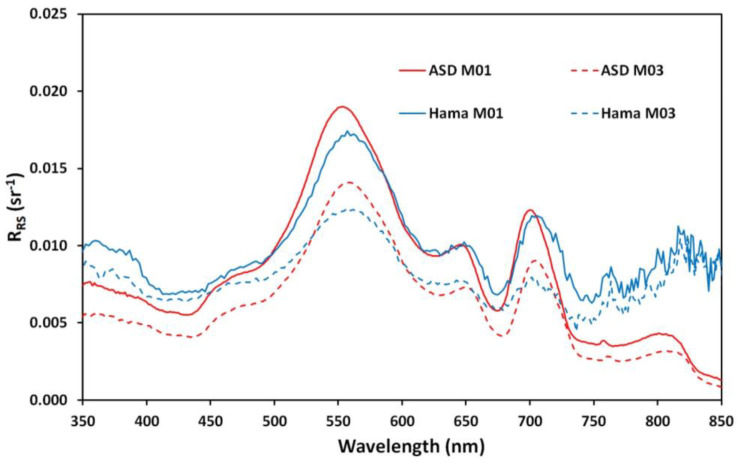
Remote sensing reflectance (R_RS_) obtained at Kelbra reservoir using the two spectro-radiometer systems, with the red lines showing ASD and the blue lines the mini-spectrometer.

The remote sensing reflectance (R_RS_) obtained with the spectro-radiometers is shown in Figure 13 and Figure 14. For Lake Süßer See (Figure 13), several spectra under stable conditions were averaged [29]. Field spectrometer data (ASD) are shown in red, and mini-spectrometer data (Hamamatsu) are shown in blue. The mini-spectrometer overestimates the remote sensing reflectance, but the spectral features are mostly identically reproduced. There is a clear stray light problem for short wavelengths, and a similar lift-off appears at longer wavelengths, but the data between 400 nm and 750 nm, which is the pivotal wavelength range for inland water RS focusing on algal growth and organic matter, appear useable.

For the Kelbra reservoir (Figure 14), which had the strong variability in surface reflectance (see Figure 13 for visible phytoplankton clusters), several dedicated measurements with and without the phytoplankton clusters were performed and averaged. The measurements without phytoplankton clusters are indicated by the solid line, and the ones with the phytoplankton clusters near the surface are indicated with the dashed line. Again, the field spectrometer (ASD) is shown in red, and the mini-spectrometer is shown (Hamamatsu) in blue. For Kelbra, a very good overlap between the averaged data of both sensors is achieved. The deviation between the two specro-radiometers is actually smaller than the deviation caused by the change in environmental parameters. The shape of the curves is again reproduced very well, with stray light problems for very short and very long wavelengths but good data in the 400 nm to 750 nm range.

#### 4.2.2. Field Test at a Freshwater Mesocosm Facility

**Figure 15:**
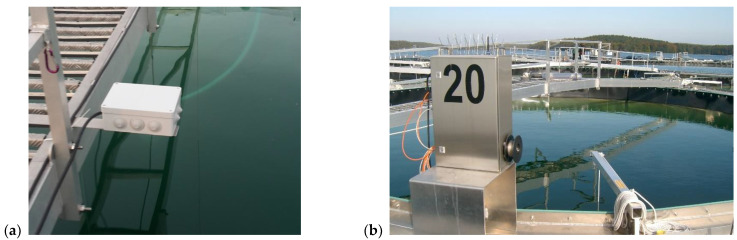
(**a**) CONNECT CoWaRa prototype v1.0 and (**b**) a mesocosm of the LakeLab facility. (Images, A. Jechow).

As a follow-up to the simple prototype tested in the campaign, the CONNECT Water Radiance (CoWaRa) prototype v1.0 was built (Figure 15a), which is designed to work permanently outdoors. For this, a weather-proof casing made from cheap components was assembled and sealed. This version still requires an external power supply. Radiometric testing was performed at a large-scale freshwater mesocosm facility [33] in comparison with another handheld field spectrometer (JETI specbos 1211UV, JETI Technische Intrumente, Jena, Germany) and with a different reflectance plate with 20% reflectivity (Zenith lite, SG3145, SphereOptics GmbH, Herrsching, Germany) [9,29]. The mesocosm measurements were performed under clear sky conditions, and due to the facility’s structure, there was only a low impact from wind, resulting in a flat surface (see Figure 15b).

**Figure 16:**
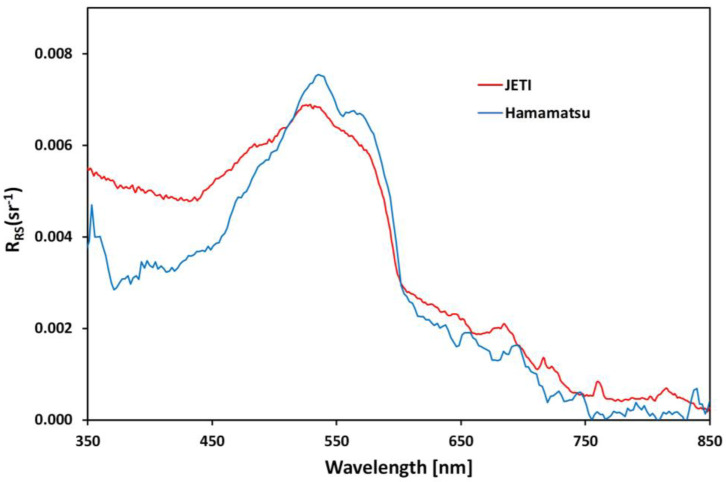
Comparison of remote sensing reflectance (R_RS_) obtained with the CONNECT WaRa prototype v1.0 compared to a handheld field spectrometer.

Remote sensing reflectance was again obtained in the Mobley geometry and is shown in Figure 16. The mini-spectrometer is plotted with a blue solid line and the field spectrometer with a red solid line. The measurements were performed manually in parallel (not with the device mounted as in Figure 15a), but small pointing errors cannot be ruled out. Despite these potential issues, the low-cost sensor resembles the spectral features relatively well, but with increased noise and some deviation at very short wavelengths.

## 5. Discussion and Conclusions

The aim of this work was to characterize and implement an affordable mini-spectrometer system, the Hamamatsu C12880MA, and test its suitability for ground-truthing applications in inland water remote sensing. Our characterization included a basic laboratory test and several field campaigns.

The mini-spectrometer performed sufficiently well in the laboratory test. The spectral resolution met our target requirements, which were a wavelength resolution of the order of 10–15 nm in the visible spectral region. The C12880MA was definitely outperformed by the expensive field spectrometer (ASD), but given its application for ground-truthing multispectral sensors, this is acceptable. The lab characterization showed some interesting asymmetry in terms of angular responsivity, and this is worth investigating for multiple devices in the future. For us, this small deviation appeared not relevant for its application in the field.

The field test clearly demonstrated the capability of the system to obtain the required spectral features of the different water bodies under several different conditions. This included the testing of different water bodies, including a mesocosm facility and two lakes, one with floating algae on the surface. While there were some deviations for short wavelengths below 400 nm and for long wavelengths above 750 nm, the deviations in the region of interest (400 nm to 750 nm) were acceptable.

Overall, the performance of the mini-spectrometer was acceptable, and the device appears to be very suitable for the targeted application. Many recent applications in other fields such as molecular trace analysis [34], crop health estimation [35] and nighttime spectro-radiometry [36] support the wide applicability of this low-cost sensor system. Such measurement systems could be integrated into automated sensor networks to monitor environmental conditions in near real-time and make the data available in information systems, for which fully automated data processing methods are beneficial [37].

The next step towards a self-sufficient system for the envisioned sensor network is the development of an independent spectro-radiometer system that can be operated in remote buoy systems. This should include (i) a battery power supply, (ii) the implementation of a sleep mode to save battery power, and (iii) a rain sensor to avoid unnecessary measurements in undesired weather conditions. Furthermore, in a future version of the system, the water-leaving radiance, the global irradiance and potentially the sky radiance should be measured simultaneously. The global irradiance system requires the use of a fiber-coupled version of the mini-spectrometer (Hamamatsu C12880MA-20) [38] with additional components or a specific solution for the reflectance plate measurements. Future applications could then include the use of the systems under cloud cover, as proposed in a recent study [13].

## Figures and Tables

**Table 1:** List of most important sensor signals of the C12880MA Hamamatsu mini-spectrometer.

Symbol	Name	I/O	A/D
CLK	Clock	I	D
ST	Start	I	D
TRG	Trigger	O	D
EOS	End of scan	O	D
Video	Video output	O	A

**Table 2:** Comparison between Hamamatsu mini-spectrometer and a standard handheld field spectrometer (ASD field spec 4).

Property	ASD Field Spec 4	Hamamatsu
spectral range	350–2500 nm	350–850 nm
spectral resolution (VIS)	3 nm	12 nm
spectral channels	2151	288
calibration	yes	no
interface	fiber optics	glass window ^1^
price	ca. 50,000 €	ca. 400 €

^1^ Optional fiber connector available.

## Data Availability

Data will be made publicly available in the future. The data are not publicly available because the CONNECT project is still ongoing and results are still being processed.

## Supplementary Materials

The following supporting information can be downloaded at https://www.mdpi.com/article/10.3390/s24196445/s1: More information on the implementation, software, and measurement protocol is available. The firmware and circuit design of the PCB board were published under the EUPL 1.2 license at https://github.com/Helmholtz-UFZ/MiniSpecFirmware (accessed on 29 September 2024) and https://github.com/Helmholtz-UFZ/MiniSpecHardware (accessed on 29 September 2024) and can be reused accordingly.
