# Supplementary material for: Characterizing and Implementing the Hamamatsu C12880MA Mini-Spectrometer for Near-Surface Reflectance Measurements of Inland Waters
Source: Sensors (Basel). 2024 Oct 5;24(19):6445. doi: 10.3390/s24196445 (PMC11479284; doi:10.3390/s24196445)
Supplement: Supplementary file 1 [file sensors-24-06445-s001.zip › sensors-3232492-supplementary.pdf]

Supplement to

Characterizing and Implementing the Hamamatsu C12880 Mini-Spectrometer for Near-Surface Reflectance Measurements of Inland Waters

Andreas Jechow, Jan Bumberger, Bert Palm, Paul Remmler, Günter Schreck, Igor Ogashawara, Christine Kiel, Katrin Kohnert, Hans-Peter Grossart, Gabriel A. Singer, Jens C. Nejstgaard, Sabine Wollrab, Stella A. Berger and Franz Hölker

Hardware implementation

To enable fast prototyping, development boards were used for the ADC and the microcontroller. In particular, this allows both components to be tested at every stage of development, and also eliminates the effort involved in layout design and implementation. The ADC development board integrates a 16 bit, 1 MSPS, successively approximating ADC2 and provides a 16 bit wide parallel interface for reading the digital data. After configuring the board, analog input signal level between 0-5 V can be digitized. The levels of the logic signals are adapted to the GPIO level of the microcontroller and correspond to 0 V (low) and 3.3 V (high). The ADC starts a digitization process when a falling edge is detected on the CNVST pin of the ADC. For the duration of the process the ADC sets a BUSY signal and with its falling edge the digital data is present at the parallel port and can be read. The microcontroller development board, hereafter referred to as the Nucleoboard, integrates the microcontroller STM32L476RGT64. It is based on an ultra-low-power ARM Cortex-M4 32-bit CPU with FPU and can be operated with a maximum clock frequency of 80 MHz. Furthermore, numerous periphery functions, such as various interfaces, FLASH and SRAM memory and timers are available. integrated in the microcontroller. All GPIOs of the microcontroller are brought out on two double pin headers of the nucleoboard. All signals are connected on a breadboard. It is plugged onto the nucleoboard and provides a connector for the sensor and for the ADC parallel port, as well as some other electrical components. Figure S1 shows a schematic diagram of the system. The schematic figure does not show the development boards and the power supply, but the 3V and 5V voltage ranges are indicated. Power is supplied to all components via the USB cable connections of the nucleoboard. At the border of the voltage ranges, level converters are used. The analog measurement signal, on the other hand, does not require any adaptation. The ADC trigger input, CNVST, reacts to falling edges, but the sensor outputs the video signal synchronously with the rising edges of the TRG signal. Therefore, an inverter is connected between both signals, which also takes over the level conversion of the TRG signal. The schematic figure shows the module names instead of the pin designations.

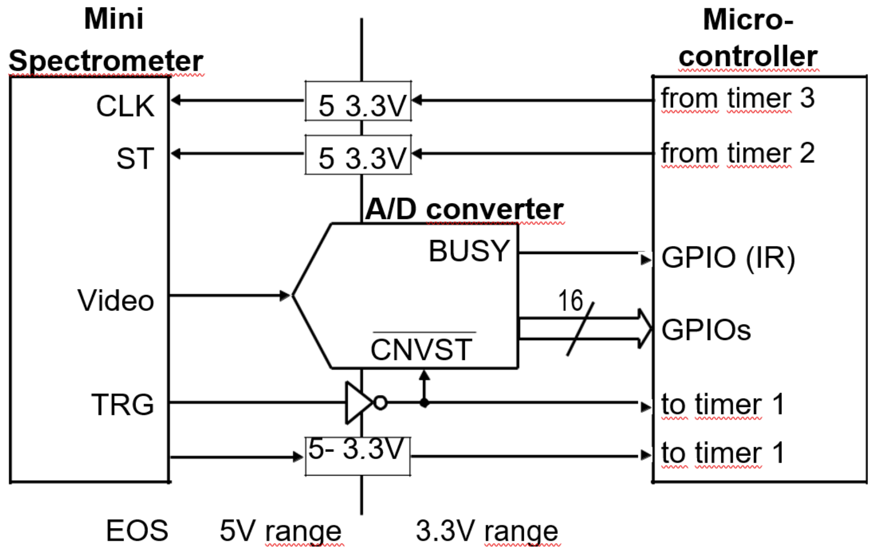

Figure S1: Circuitry of the prototype. The voltage supply and the Development boards are not shown. IR: Interrupt

Firmware implementation

The firmware is written in the C programming language and was developed with the integrated development environment AC67 . Furthermore, the gene-rator for initialization code CubeMX8 was used, as well as a hardware abstraction layer (HAL) and libraries provided by ST. An extended program flow chart in Figure S2 gives an overview of the firmware. There above all the program flow of the main program, briefly main() and the measuring sequence is represented in detail, since these form the core of the firmware. The measuring sequence is a subroutine of the main(). Additionally the interrupt routines (ISR) and the timer modules are shown, whose relations to the regular program flow are indicated by dotted lines. In the following, the main program flow is explained first, then the measurement routine and finally the communication with the system, the protocol. The sequence of a measurement is controlled to a large extent by four timers. For a simplified description limited to the functions used, please refer to the timer subchapter below.

Main program

The execution starts with the entry into the main() function. Here first the HAL and the used microcontroller modules are initialized. The latter include the clock generation, the GPIO, and the UART module as well as the timers and the interrupt controllers, EXTI and NVIC. Then the receiving of the UART module is activated. In doing so, it receives characters independently and is also configured to store them in memory via direct memory accessff (DMA). Since the UART module does not require any CPU interaction, it can operate completely independently, especially in sleep mode. Once initialization is complete, execution enters an infinite loop. In the loop the character recognition interrupt of the UART module is activated. Thus the reception of a Carriage Return10 (CR) character triggers the interrupt and the execution jumps into the corresponding interrupt handling, in the following called CR-ISR. In the CR-ISR a variable is set, which marks the possible reception of a command, because the CR serves as termination character of the commands. The next step in the loop is to check whether a CR has been received. If no CR was received, the CPU switches to sleep mode. Thereby it is ensured by means of different methods11 that the execution stops and only the receipt of a CR character activates the CPU again. If a CR was received, the CR interrupt is deactivated in order not to disturb the further execution, especially not to influence time-critical processes in the measuring sequence. Then the command parser is called. It searches the UART receive puffer for valid commands and sets a global variable (extcmd) according to the command found. If no command is identified extcmd is CMD_UNKNOWN. In the latter case the loop is finished and starts from the beginning, otherwise the execution branches based on extcmd and the corresponding action is executed. If an action was successfully executed, the loop pass is completed and starts from the beginning. However, some actions may fail and return an error code. In this case, an error handling routine is called before the loop ends. It returns the possibly corrupted system to a defined state and informs the command sender about the failure of the last command. Note that the CR interrupt is disabled during active execution. If the UART module receives a CR during this time, it still sets an interrupt flag in its interrupt status register. When the CR interrupt is reactivated, it occurs immediately and is handled (CR-ISR). Therefore, before entering sleep mode, it is checked whether a CR has already been received. The stream command represents a special case in the program sequence, since it changes the regular sequence. After receiving the stream command, the CPU is no longer set to sleep mode in all following loop passes. To return to the regular sequence a stream terminate command is available. This special case is not shown in the program flow chart.

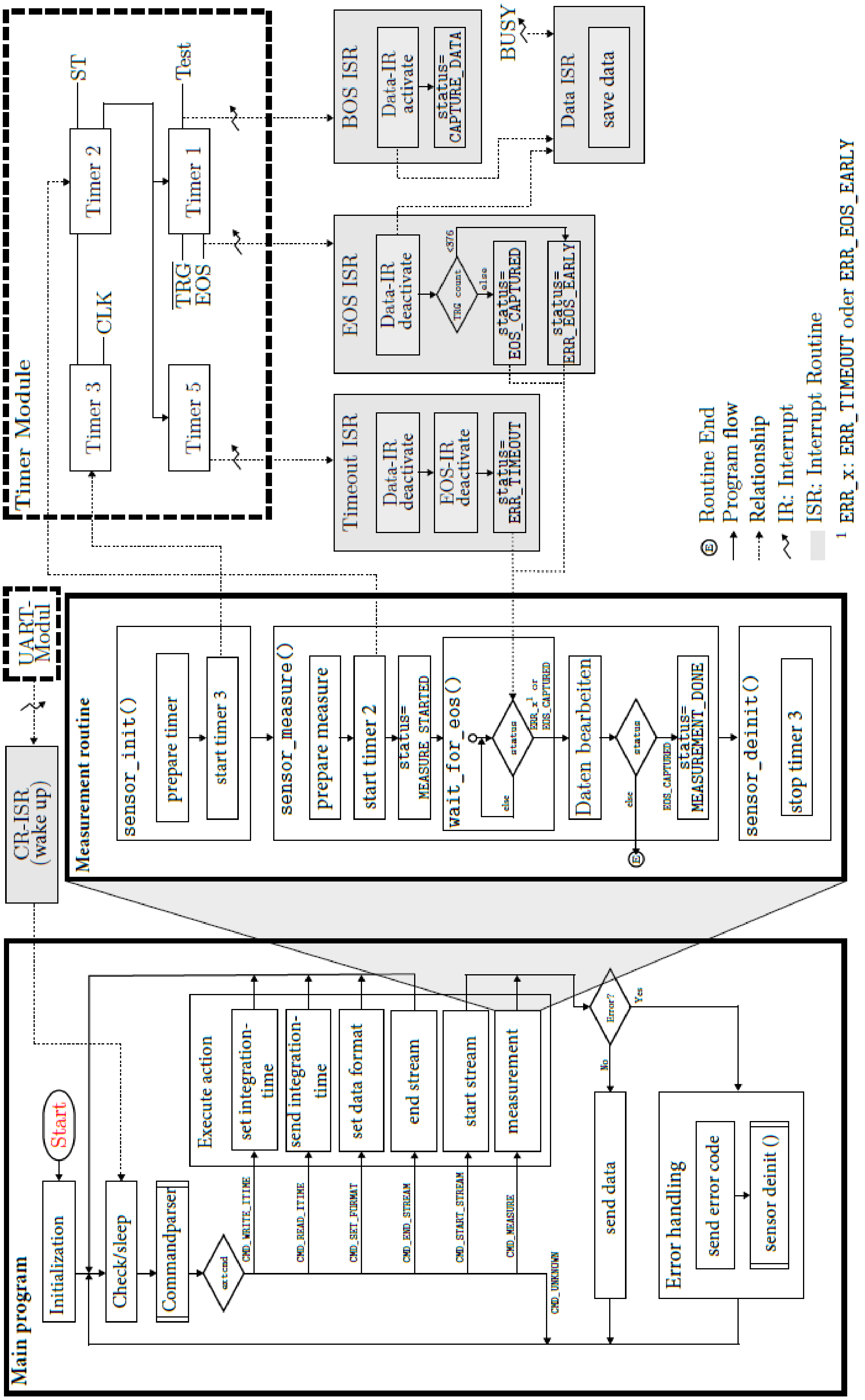

Figure S2: Overview of the firmware as an extended program flow chart. In addition to the program sequence, the most important relationships are also shown. BOS: Start-Of-Store. EOS: End-Of-Scan.

Measuring routine

The user system initiates a measurement by sending a command, whereupon the sensor_init() functions is called. It initializes the timers and provides a memory area for the measurement data. Then it starts timer 3 and changes the status of the measurement to INITIALIZED. After initialization the function sensor_measure() is called, which sets the timer settings for the measurement. In particular, the value for the ST pulse duration is written to timer 2 to obtain the desired integration time. Then timer 2 is started and the status is set to MEASURE_STARTED. This starts the actual measurement, which is controlled exclusively by the timers and ISRs. To continue the execution after the measurement, the sub-function wait_for_eos() is called. It waits for the end of the measurement by repeatedly polling the status. Figure S3 shows the interconnection of the timers and Figure S4 shows the control signals which they generate and to which they react. Timer 3 This timer generates two output signals, one of which feeds the clock input of the sensor and one of which feeds Timer 2. The signals in both cases a 1 MHz clock with a 50% duty cycle, but they are offset from each other by shifted by a quarter period. The shift ensures that the edges of the ST signal are always midway between the falling and rising edges of the CLK signal - as shown in Figure S4(b). Thus, a defined counting of the TRG pulses can be achieved. Timer 2 This timer generates the ST signal for the sensor and a trigger signal for the remaining two timers. With the falling edge of the ST signal, the internal timer trigger output (TRGO) changes from low level to high level and activity. The timer 2 deactivates itself at the same time (one-pulse mode). Timer 5 Like Timer 2, this timer uses the one-pulse mode and deactivates after 500 µs. It serves as a safety timer12 and prevents a deadlock of the program in case of an error. After expiration it sets the status to the error value ERR_TIMEOUT and deactivates all other timers. After successful measurement Timer 5 in sensor_deinit() disabled to avoid false timeout. Timer 1 This timer performs four tasks. It counts the TRG pulses, generates the test signal, activates the saving of the measured values and detects the EOS signal. Its clock input is fed by TRG and is inverted internally. Thus, in simplified terms, its count register reacts to rising edges of the TRG signal. If the counter register reaches the value 85, the CCR2 triggers the BOS13 interrupt and the BOS-ISR is called. It activates the data interrupt and sets the status to CAPTURE_DATA. Simultaneously to the BOS interrupt the output logic of the CCR2 reacts and sets the test signal to the high level. If timer 1 detects the EOS signal, the EOS interrupt is triggered and at the same time the value of the counter is stored in the CCR4. The associated EOS-ISR deactivates the data and the EOS interrupt and then checks whether the value of the CCR4 corresponds to the desired number of TRG pulses. If the value is less than 376 the status is set to the error value ERR_EOS_EARLY, otherwise to EOS_RECEIVED14. Data interrupt The data interrupt is a simple GPIO interrupt, which responds to rising edges of the BUSY signal. In the data ISR, a check is first made to see whether the data Puffer is already full, in order to prevent a Puffer overflow. If data can still be stored, the input values of 16 GPIOs are read and written to the Puffer. The 16 GPIOs are connected to the parallel port of the ADC. Finally the write pointer of the Puffer is incremented. End of a measurement The status corresponds either to an error value or EOS_RECEIVED. In either case, execution in wait_for_eos() is terminated and post-processing of the data follows. The bits of each data word are re-sorted, because they are not in the correct order. Since a memory area was provided for the data even in the case of an error, branching before post-processing is not necessary. If the measurement was successful, the status is set to MEASUREMENT_DONE and the measurement routine is finished. In case of error the status remains unchanged, because it contains information about the error and is evaluated in the main program.

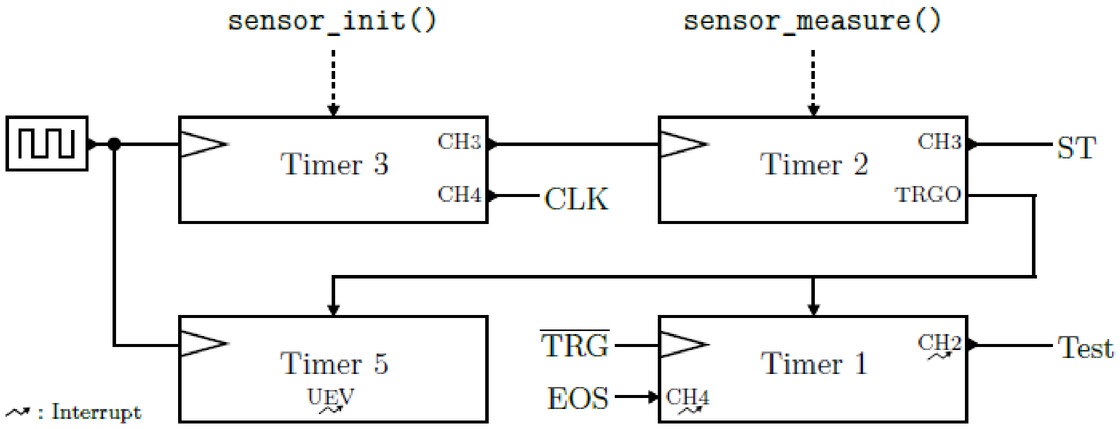

Figure S3: Wiring of the timers used. Inputs are shown on the left, outputs on the right. Arrows show the activation of the timer. The interrupts have the following effect: At the same time as the rising edge of Test, the saving of the measured values is activated. The counter overflow/update event (UEV) in Timer 5 and a detected edge at EOS deactivate saving again. TRGO: Timer trigger output

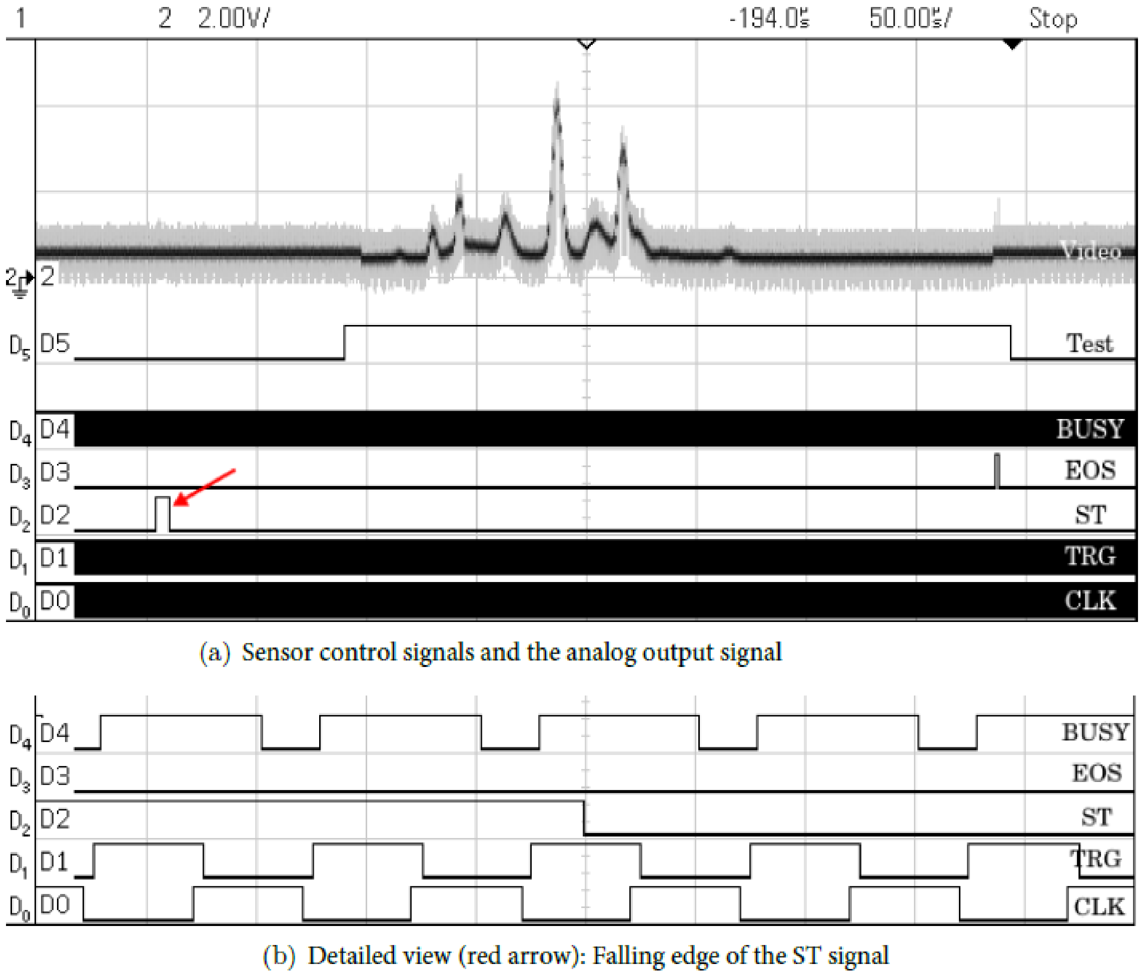

Figure S4: Control signals that the timers generate (CLK, ST) and to which they react (EOS, TRG). The BUSY signal is processed by the data ISR.

Protocol

The commands and associated actions defined by the protocol so far are listed in table S1. Depending on the selected format, the arguments of the commands are transmitted ASCII or binary coded, where the byte order of all binary coded values is little-endian. The returned data is also transmitted in the same format, but the commands themselves are always ASCII-encoded. The last character (including argument) of a command must always be the CR character, otherwise the command will not be recognized by the parser (see also footnote 10). A measurement data packet consists of 578 bytes. In the first place there are 2 zero bytes, then 288 16bit data words follow. An error code, which is sent in case of an error, is a single 16bit data word. The possible error codes are shown in table S2. The measure command starts a measurement and the measurement routine is called as described in the previous subchapter. After successful measurement a data packet is transmitted to the command sender, in case of error only a corresponding error code. The stream command starts a continuous measurement. A measurement routine similar to the measure command is executed. After successful measurement the data packet is transferred. After the transmission, another measurement starts immediately. If an error occurs in one of the measurements, the corresponding error code is transmitted and the stream terminates itself. With the commands format= and itime= the arguments are checked by the parser. If itime= detects an integration time outside the valid interval, the integration time is set to the maximum or minimum value, depending on whether the interval was exceeded or not. With format= the format is set to ASCII in case of an invalid argument.

Table S1: Protocol: Commands

**Command**	**Argument**	**Return**	**Description / Action**
measure	-	Data package	Starts (single) measurement
stream	-	Data packets	Starts continuous measurement
end	-	-	Ends continuous measurement
format=	0/1	-	Sets the data / argument format<br>0: binary, 1: ASCII
itime=	54...1000000	-	Sets the integration time in µs
itime?	-	54...1000000	Sends the set integration time (µs)

Table S2: Protocol: Error codes

**Error code**	**Description**
0x0000	No error, 576 bytes of data follow
0x0001	Unknown error
0x0002	(obsolete)
0x0003	EOS signal detected, but not all data of the sensor received
0x0004	Timeout - No EOS signal after integration time + 500 µs

Laboratory examination

The following experiments were performed in the laboratory:

E-1 Dark current of the sensor

E-2 Angle dependence of the sensor horizontally

E-3 Angle dependence of the sensor vertically

E-4 Repeatability of the sensor

E-5 Sensitivity / resolution of the sensor

E-6 Radiation intensity of the source used

In the following, first the general setup and the instruments used are briefly described, then the experiments themselves and their execution are explained. Finally, the most important methods of data evaluation are explained.

Preparation and experimental setup

The setup for the laboratory tests is shown in Figure S5. In a closed darkening box, a receiver (for E-1 to E-5 the sensor, for E-5 the sensor) is placed. E-6 a photometer (15S120VC, Thorlabs GmbH, Dachau, Germany) and a collimator (16NA= 0.51, f = 7.86 mm) are mounted. The collimator is connected to the output of a monochromator (17MSH-300, LOT-QuantumDesign GmbH, Darmstadt, Germany) via an optical fiber. The receiver and the collimator are each mounted on a base on a rail to avoid a change in position. The output slit of the monochromator is set to 1.48 mm, so that a peak with a full width at half maximum of 4.00 nm is achieved at the output (E-5, E-6). The sensor is mounted in a holder on a stepper motor. The holder has two holes offset at right angles to each other and thus offers two alignment positions of the sensor on the stepper motor. The window and in particular the input slit of the sensor underneath is oriented once horizontally and once vertically towards the light source. The experiments E-2 and E-3 are also named after the position of the slit. The stepper motor can be rotated horizontally in 0.9° steps. The zero angle indicates the aligned starting position - sensor and collimator are opposite each other - as shown in figure S5. In the mathematical positive direction of rotation, the sensor is turned away from the observer, in the negative direction towards the observer. The control of all components is automated and controlled by a LabView (LabVIEW, National Instruments, Version 9.0) script from a conventional PC. The monochromator, the step motor driver and the photometer are integrated into LabView by prefabricated instrument classes (VIs), which are provided by the manufacturers. The serial interface for the sensor is provided by a TTL-USB converter. The data, which are recorded during the measurement, are stored by LabView as CSV files. A detailed description of the control by the LabView code is omitted, since the procedure is evident from the descriptions of the experiments.

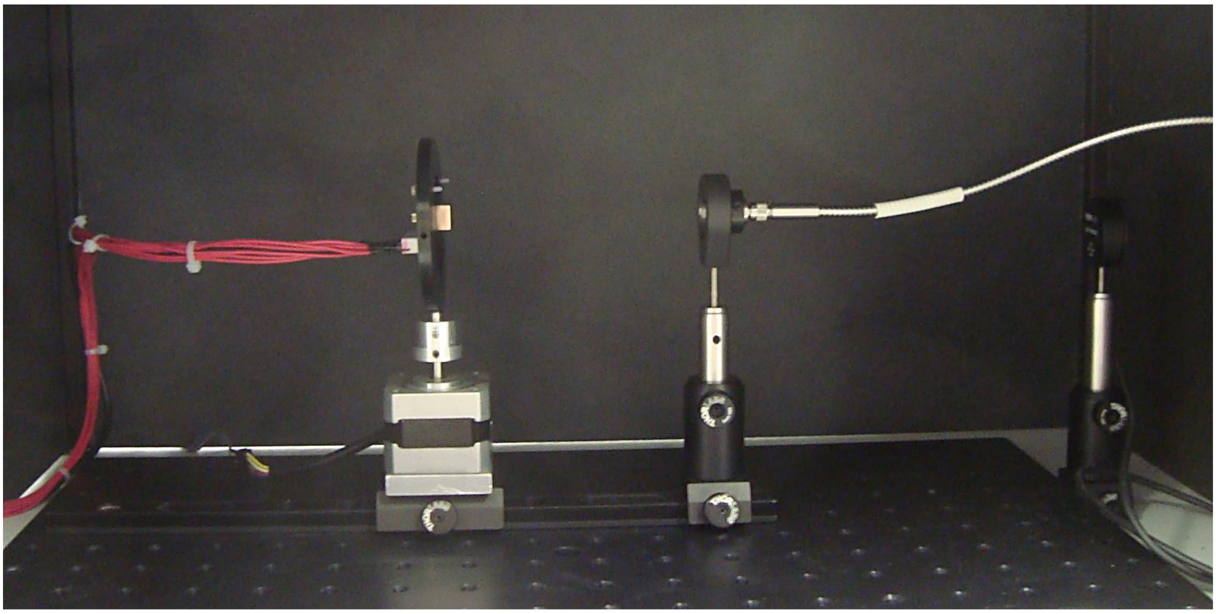

Figure S5: Experimental setup in open darkening box a) Sensor on stepper motor stepper motor b) Collimator at the output of the optical fiber c) Photometer (for experiment E-6)

Implementation

At the beginning of each examination, the alignment of the pedestals for the respective receiver in distance, height and angle was carried out, whereby in some cases positions were marked to ensure a quick conversion. The darkening box was then closed and all avoidable and unnecessary radiation sources were switched off. Then the respective measurement took place, the results of which are referred to as measurement data packages. A measurement data package contains one value per pixel.

E-1 The first experiment is to record the dark current of the sensor to be able to determine the Offset per pixel present in each measurement. For this purpose, the sensor input was covered in the darkening box and the monochromator did not emit any radiation. 146 measurement data packets were recorded.

E-2, E-3 In these experiments, the angular dependence of the sensor is to be determined. Both experiments are identical in execution, only the position of the sensor in its holder varies. In the implementation, a broadband spectrum was output from the monochromator and this was measured for different angles. The angles from -45° to 45° were measured in steps of 0.9°. Afterwards, the range was traversed in the same retrograde manner. Both directions were repeated once. This resulted in a total of 400 measurement data packages per experiment.

E-4 For the analysis of the repetition accuracy in this experiment, the measurement setup was set up, four measurements were recorded and dismantled again. 40 repetitions were performed, resulting in 160 measurement data packets. The monochromator outputted a broadband spectrum. The goal of this analysis is to determine a percentage error that can be expected when comparing spectra.

E-5 This experiment is used to determine the resolution and sensitivity of the sensor. Since both properties are wavelength dependent, measurements must be taken at different wavelengths. Therefore, the wavelength range from 350 nm to 860 nm was traversed by the monochromator in 1 nm steps and a measurement data packet was recorded for each step. The monochromator output a peak with a full width at half maximum of 4 nm at each step. The experiment was repeated four times (2200 measurement data packages).

E-6 This experiment serves to characterize the radiation intensity of the monochromator of the previous experiment. This is because the intensity output in each case is also wavelength-dependent. To determine it, the same spectra (or peaks) are generated as in the previous experiment E-5, but instead of a measurement data packet, the intensity value is recorded with the photometer.

Evaluation methods

The measured values output by the sensor do not represent absolute intensity, but relative values to each other. Therefore, the values are unitless and are called count or ADC-count. The value of a single pixel i is denoted by c_i, $i \in 1, 2, ..., 288$. If the values of all 288 pixels are meant, the total unit is called pixel spectrum and is symbolized by c. If wavelengths are assigned to the pixel numbers, c_λ denotes the intensity value at the corresponding wavelength λ. The center wavelength output by the monochromator is denoted by λ_M. If, on the other hand, the wavelength is calculated from the pixel numbers (see next section), λ_H is used. The manufacturer provides a 5 order polynomial for each mini spectrometer, which is used to calculate the central wavelength assigned to a pixel. For the used sensor (C12008MA-15B00039) the following polynomial was provided:

$$H_{korr}(x) = a_0 + b_1 \mathrm{x} + b\, x_2{}^2 + b\, x_3{}^3 + b\, x_4{}^4 + b_5\, x^5 \qquad (2.3)$$

$$\begin{aligned} \text{with } a_0 &= 3.175\,107\,897 \times 10^2 \\ b_1 &= 2.683\,434\,727 \\ b_2 &= -8.912\,972\,570 \times 10^{-4} \\ b_3 &= -1.001\,126\,990 \times 10^{-5} \\ b_4 &= 1.813\,212\,164 \times 10^{-8} \\ b_5 &= -7.003\,857\,032 \times 10^{-12} \end{aligned}$$

A dark current correction value is determined for each pixel of the sensor from the data of experiment E-1. The full width at half maximum as a measure of spectral resolution is to be determined from the data of experiment E-5. Since the sensitivity of the sensor is wavelength dependent, an approach to determine a correction function shall be given. For this purpose, the data of the sensitivity measurements E-5 are used. The added value is interpreted as the intensity of the recorded monochromator peak. This value is subject to a distortion due to the sensitivity of the sensor. Since the correct (normalized) value of the intensity is known, the distortion can be determined.
